# Development of a Poisoned Bait Strategy against the Silverfish *Ctenolepisma longicaudata* (Escherich, 1905)

**DOI:** 10.3390/insects11120852

**Published:** 2020-12-01

**Authors:** Anders Aak, Morten Hage, Heidi Heggen Lindstedt, Bjørn Arne Rukke

**Affiliations:** Department of Pest Control, Norwegian Institute of Public Health, Lovisenberggata 8, P.O. Box 222, Skøyen, NO-0213 Oslo, Norway; anders.aak@fhi.no (A.A.); morten.hage@fhi.no (M.H.); heidi.lindstedt@fhi.no (H.H.L.)

**Keywords:** *Ctenolepisma longicaudata*, control, demography, efficiency, poisoned bait, population collapse, safe strategy

## Abstract

**Simple Summary:**

*Ctenolepisma longicaudata* is emerging as a nuisance pest in private homes in some European countries, and it is considered a serious problem in museums and libraries where it can do damage to objects of historical value. It is a silverfish that may be difficult to eradicate because it utilizes many parts of a building. Heavy use of pesticides is undesirable from a health perspective, and baits with low concentrations of toxins are consequently preferable. To safeguard the indoor environment during management, the present study describes procedures for the efficient control of *Ctenolepisma longicaudata* with small amounts of bait. This is as efficient, as sprayable pesticides and declines with more than 90% reduction of the pest population can be achieved within 15 to 20 weeks. Successful eradication can be achieved with as little as 0.5 to 1.0 g bait per 100 m^2^.

**Abstract:**

Pest management strives to be an efficient, yet healthy and environmentally safe control method, and the use of poisoned bait often fulfils these criteria. In the present study, we show that bait with indoxacarb as the active ingredient is highly efficient for controlling *Ctenolepisma longicaudata* (Escherich, 1905) and two of its relatives, *Lepisma saccharina* (Linnaeus, 1758) and *Ctenolepisma calva* (Ritter, 1910). Applying small bait droplets (size ~10 mg) along the walls of several types of buildings, at no more than 0.5 to 1.0 g bait per 100 m^2^, was a cost-efficient and safe strategy for the knockdown and eradication of *C. longicaudata*. During field experiments, the demography changed from an initial mixture of different stages to total dominance of early instars preceding the population collapse. Poisonous bait outcompeted mass-trapping with sticky-traps and conventional insect spray treatment for the efficient control of *C. longicaudata* in apartments. Different droplet densities (1 vs. 0.5/m^2^) and active ingredients (indoxacarb vs. clothianidin) did not have different effects in field experiments. These results show that poisoned bait is a highly relevant tool for managing *C. longicaudata* and potentially against other silverfish infestations.

## 1. Introduction

The long-tailed silverfish, *Ctenolepisma longicaudata* (Escherich, 1905), (Zygentoma: Lepismatidae) is an indoor pest encountered on most continents [1,2,3]. Silverfish are considered a problem in museums and libraries, as they consume paper and other plant-based materials [4,5]. *C. longicaudata* may also affect private homes where objects of value are at risk, but no structural damage occurs to the building, as it is mainly considered a nuisance pest [3,6]. However, recent studies in Europe have highlighted that this species is particularly troublesome in modern buildings [6,7,8] where it can reach high densities and spread between rooms and apartments [6]. Adult *C. longicaudata* reach a considerable size comparable to adult German cockroaches (13–18 mm [9,10]). This causes mental distress among many homeowners because high densities of large, swiftly crawling insects are undesired indoors. An increasing number of observations of this species in Europe [10,11,12,13,14,15,16] indicates that this silverfish species is an emerging indoor problem and warrants the development of an efficient and safe control method.

Indoor living lepismatids are regularly encountered. *Thermobia domestica* (Packard, 1873), the firebrat, is commonly present in warmer regions, and *Lepisma saccharina* (Linnaeus, 1758), the common silverfish, is frequently observed under moist indoor conditions worldwide [1,2,3]. Both species can be treated using simple and cost-efficient control efforts, whereas *C. longicaudata*, *Ctenolepisma lineata* (Fabricius, 1775), the four lined silverfish, and the co-occurring occasional pest *Ctenolepisma calva* (Ritter, 1910), are considered more difficult to eradicate as they often have a building-wide and more uniform spatial distribution [2,6]. The latter three species are more drought tolerant and can survive and reproduce at relative humidity levels commonly found indoors [1,17]. This ability combined with a life cycle of 13 immature stages and requiring one and a half to two years for completion at 22 °C [17] extends their period of proliferation to three to four years before detectable density levels are reached [6]. As several parts of a building are utilized by these species, control efforts require a wider spatial reach and consequently become more labor demanding and costly [1,2]. A traditional control situation requires preparing the premises by reducing the humid conditions and vacuum cleaning to remove dead insects, leftovers, and vegetable litter that may act as food sources [2]. Cracks and crevices, as potential harborages, require treatment with insecticides to kill hidden individuals. Such a building-wide application of pesticides is undesirable from a health perspective and should be avoided, as toxins degrade slowly indoors and may produce a chronic exposure risk for the residents [18,19,20].

Poisonous bait (hereafter denoted as bait) is generally considered a safer control method compared to sprayable pesticides [21,22] and has been applied successfully against cockroaches [23,24,25,26,27,28]. Although silverfish and cockroach biology have many common features, bait is considered inadequate for controlling silverfish. This is most likely due to the slow rate of mortality with LT_50_ values > 9 days [29], or may stem from a meticulous life and broad distribution causing the field effects to manifest slowly [2,6]. However, recent laboratory studies have highlighted bait as a potentially efficient approach towards control [30]. Several active ingredients in currently available commercial bait cause high levels of initial mortality in *C. longicaudata*, and indoxacarb evokes as much as 75% secondary mortality through the consumption of dead, poisoned conspecifics [6,30]. *C. longicaudata*, the main culprit in Norwegian homes [6], and *T. domestica* feed willingly on dead conspecifics, feces, and shed skins [17,30,31] which may promote secondary poisoning and elevate the effect of the bait under field conditions. Bait appears to maintain its functionality for as much as six months even if it dries out [30]. In total, this indicates a strong potential for a bait strategy, and when combined with a life cycle of several instars that require multiple feeding events before maturation, a strong population impact is expected. No long-term full scale field experiments have been conducted to evaluate bait strategies against *C. longicaudata*, but a case-study conducted in Holland described promising effects from the use of bait with clothianidin as the active ingredient [16].

We conducted a series of field experiments in Norwegian homes, businesses, and public buildings to test bait as a control method against *C. longicaudata* and to evaluate the possibility of total building-wide eradication. The aim was to understand the mechanisms behind potential population declines and to describe the cost efficiency of the control efforts. We present the results of field comparisons between a spray application (permethrin), mass trapping (sticky traps), and bait (indoxacarb), as well as differences in bait placement and a comparison between two active ingredients (indoxacarb vs. clothianidin). Finally, we evaluate seven full-scale control cases through declines in population densities and demographic changes in the pest populations.

## 2. Materials and Methods

The field studies were conducted in Oslo and Viken counties in Norway between 2017 and 2020. Since 2016, this area has experienced a strong increase in infestations of *C. longicaudata* [6], and relevant control sites were selected through the Norwegian Institute of Public Health’s contact network of pest control technicians, insurance companies, and official institutions. In parallel with the field studies, we also conducted laboratory investigations that indicated improved efficiency by using a dispersed, as opposed to a more concentrated, distribution of small droplets of bait [6]. Thus, the bait stations used initially were abandoned in favor of direct bait application.

Most of the field experiments were evaluated with a fixed number of commercial monitoring traps (Trapper monitor and insect glue traps, Killgerm, Ossett, UK or Silvalure–window insect monitors, Silvanderson, Knäred, Sweden) in fixed positions at each locality for 14 days, and we scored the number of insects caught per fortnight. Measures of population densities were always taken before placing the bait, and at four to nine subsequent fortnights. Most experiments were terminated when the trap count in the building reached zero.

The Advion^®^ Cockroach (0.6% indoxacarb, Syngenta, Basel, Switzerland), Advion^®^ Ant (0.05% indoxacarb (neurotoxin; blocking of sodium channels), Syngenta) and Maxforce^®^ Platin (0.5% clothianidin (neurotoxin; activation of post-synaptic acetylcholine receptors), Bayer, Leverkusen, Germany) baits were used as described below. Experiment 1 compared bait with two other control strategies. Experiment 2 compared variation in bait droplet density. Experiment 3 evaluated two different active ingredients. Experiment 4 investigated field efficacy in seven different indoor environments (location 1–7). At the two largest sites (Experiments 2 and 4 (location 6)) we also evaluated the practical aspects of a bait strategy by quantifying the amount of bait used and the time spent conducting the job.

Demographics were investigated by grouping captured individuals according to size. Two groups of small individuals were discerned by the presence or absence of scales, such as nymphs without scales (stages 1–3) and nymphs with scales (stages 4–8), in addition to maturing nymphs with partially developed styli (stages 9–13) and adults with fully developed reproductive organs (stage 14 and above).

Experiment 1, three different control strategies: 30 apartments in a 154-apartment complex were assigned to either permethrin spray, bait, or mass trapping. Care was taken to ensure similarity among the apartments in terms of size, number of residents, tidiness of the apartment (subjectively scored), dirt present (standardized and timed vacuum cleaning to equalize the apartments), indoor conditions (temperature and relative humidity), and initial *C. longicaudata* population density (Table 1). The numbers of each treatment were 11, 10, and 8 apartments for sprayable pesticides, bait, and mass trapping, respectively. Including a control treatment for comparison would have allowed a more precise quantification of the different effects, but we were unable to recruit such a group of volunteers, as all residents with infestations were determined to get rid of the nuisance. The effect from the three treatments was evaluated by monitoring traps (Trapper monitor and insect glue traps) at a density of 0.3 traps/m^2^ positioned alongside walls in a continuous fortnight-series lasting for 10 weeks.

Mass trapping was conducted by adding an average of 40.1 ± 3.7 sticky traps (Trapper monitor and insect glue traps) with cricket powder (100% *Acheta* cricket, Unik mat–Asker, Asker, Norway) as an attractant [6] to the monitoring traps already present in the apartments. The traps were evenly distributed throughout the apartment beneath large furniture or along walls. Because residents found these additional traps more annoying than the *C. longicaudata* problem, the mass trapping attempt was terminated after four weeks.

Permethrin (Trinol Super Permetrin—9.5 g/kg Permethrin, 3.4 g/kg Pyrethrin II and the synergist piperonyl butoxide 15.1 g/kg) was applied according to label instructions by spraying all available cracks and crevices in wall skirtings or door frames and cavities under fixed furniture such as cabinets, book shelves, refrigerators, dishwashers, and washing machines. The spray was therefore mainly directed towards potential hiding places and their surrounding surfaces to minimize pesticide use indoors. An average of 105.9 ± 13.1 g (approximately 1 dl of aerosolized liquid) of the product was used per apartment. Residents were instructed to avoid washing away the pesticides during the first six weeks.

Advion^®^ Ant bait was applied in surplus at an average of 25.7 ± 1.9 bait stations per apartment. Bait stations were made from Trapper monitor and insect glue traps with the sticky surface covered, and bait was deposited inside the trap together with cricket powder as an attractant [6]. The bait and attractant were replaced twice during the 10-week evaluation period.

Experiment 2, effects of high or low density bait droplets: bait was chosen as a building-wide control approach in the 154-apartment complex. The bait experiment was conducted for 10 months after completing the test of the three different control strategies (Experiment 1). The apartment complex consisted of four similar high-rise buildings connected through an underground parking house. Two of these buildings received a high density of bait droplets (droplet density: 1/m^2^, per apartment) and the other two received a low density (0.5/m^2^, per apartment). The bait droplets were placed along walls in the apartments, and in cracks and crevices where possible. Technicians used Advion^®^ Cockroach bait, and each apartment was treated three times at intervals of two to four months. The droplet size was approximately 10 mg, and the technicians used 15 or 10 min and 0.55 or 0.30 g per high- and low-density apartment, respectively (Table 2). A total of 31 apartments, such as seven to nine apartments evenly distributed in each high-rise building, was followed closely for the effect-evaluation by monitoring traps (Trapper monitor and insect glue traps) at a density of 0.2 traps/m^2^ during six fortnights at four to 14 week intervals.

Experiment 3, comparison between two different active ingredients: The four high-rise apartment buildings investigated for high or low density of bait droplets were connected by a row of 12 two-story townhouse apartments. The entire row of townhouses was treated, and every second apartment was assigned to either Advion^®^ Cockroach or Maxforce^®^ Platin to compare baits with different active ingredients. The apartments were treated three times, two months apart with a high density of droplets (1/m^2^), and were effect-evaluated by monitoring traps (Trapper monitor and insect glue traps) at a density of 0.1 traps/m^2^ during seven fortnights at two to 13 week intervals. Three residents could not participate in the monitoring program, leaving the balance at four vs. five apartments for the Advion^®^ Cockroach and Maxforce^®^ Platin treatments, respectively.

Experiment 4, field efficacy at seven different locations: seven different localities were treated with bait. The control strategy varied somewhat by using either Advion^®^ Cockroach or Advion^®^ Ant, the number of bait applications, the density of the droplets and small discrepancies in application strategy due to local safety considerations, but was generally conducted by applying a large number of bait droplets throughout the building/locality followed by an evaluation period to quantify the reduction in the population (Table 3).

Location 1: Five individually separated commercial areas at street level (1800 m^2^; café, hairdresser, grocery store, pharmacy, and a health clinic) were treated with Advion^®^ Cockroach once and effect-evaluated with traps (Silvalure–window insect monitors) placed along walls at locations with limited customer activity to hide them from view and to reduce the destruction or removal of the traps.

Location 2: There was a large underground common area underneath the commercial zone, with distinct sectors made from parking lots, storage rooms, and interconnecting halls and stairways. This common area was treated with Advion^®^ Cockroach twice and effect-evaluated with traps (Silvalure–window insect monitors) placed along walls at locations with limited resident activity to prevent the destruction or removal of the traps.

Location 3: An apartment complex with 37 apartments over seven stories was treated with Advion^®^ Ant twice, and then once more in individual apartments if *C. longicaudata* was observed during the latter part of the evaluation period. In addition to bait, we used cricket powder as an odor stimulant [6] placed in a deactivated single sticky trap in each room. This stimulant was intended to increase food searching behavior and contact probability with the bait. The treatment was effect-evaluated with traps (Silvalure–window insect monitors) in 16 of the 37 apartments.

Location 4: A four-unit row house was treated with Advion^®^ Ant three times. Because the households included small children and pets, most bait droplets were placed at hidden locations alongside the walls, underneath skirtings, and behind furniture. An approximation of high density was attempted, but the droplets were unevenly distributed in some places considering the children and pets. The apartments were effect-evaluated by monitoring sticky traps (Trapper monitor and insect glue traps) evenly distributed in all four units.

Location 5: A three-unit row house was treated with Advion^®^ Cockroach once. Additionally, after 10 weeks, we placed one Advion^®^ Cockroach bait station (0.5% indoxacarb, Syngenta, CH) per 4 m^2^ that remained throughout the experiment to counter any potential limitations of droplet quality or removal during cleaning. The apartments were effect-evaluated by monitoring sticky traps (Trapper monitor and insect glue traps) evenly distributed in all three units.

Location 6: A 35,000-m^2^ research facility with four large buildings was treated once with 360 g Advion^®^ Cockroach. Bait was only used at locations accessible without moving large furniture and was mainly placed in small cracks underneath floor skirtings or behind other objects to avoid removal during cleaning of the facilities. The effect was evaluated with traps (Silvalure, window insect monitors) in 47 pre-selected laboratories, offices, storage rooms, wardrobes, and toilets.

Location 7: A kindergarten building hosting four children sections was treated with Advion^®^ Cockroach. The bait was applied twice during two holidays, five weeks apart. The bait was applied on the masking tape along skirtings in play areas and was removed after five days during the first holiday and after 22 days during the second holiday. Bait was placed permanently underneath skirtings in areas inaccessible to children, such as storage rooms, offices, and basements. The kindergarten was effect-evaluated by monitoring sticky traps (Trapper monitor and insect glue traps) evenly distributed in all sections, but only in place during weekends when the children were absent.

*Ctenolepisma calva* was detected in some of the apartments (Experiment 2) and *Lepisma saccharina* was observed in laboratories, wardrobes, and toilets at the research institute (Experiment 4, location 6). The effect of the bait on these populations was measured with the same traps and time schedule as described above, but only apartments or rooms with the species were included in the effect evaluation.

Statistical analyses: Statistical analyses were conducted with SigmaPlot for Windows version 14.0, build 14.0.0.124 (Systat Software, Inc. La Jolla, CA, USA). We used repeated measures analysis of variance (rm-ANOVA) with apartment/room/zone as the subject, time as the level of treatment, and catch number as the response variable. If normality tests failed, we used rm-ANOVA for ranks. Dunn’s method was used as a post-hoc test for all pairwise multiple comparisons. All averages are given with standard errors (±SE). The relative numbers according to initial density are used to ease comparison between the seven different localities (Experiment 4) and highlight the similarity of the general impact of the bait against *C. longicaudata*. Two apartments with missing data points were excluded from experiment 2, as rm-ANOVA for ranks requires a complete dataset. The measures from the café and the next-door hairdresser in the commercial area were pooled due to owner changes and renovation causing limited access. The research institute was not included in the evaluation of stage-specific effects, as only the total numbers of insects caught in the traps were registered as the initial measurements.

## 3. Results

**Figure 1:**
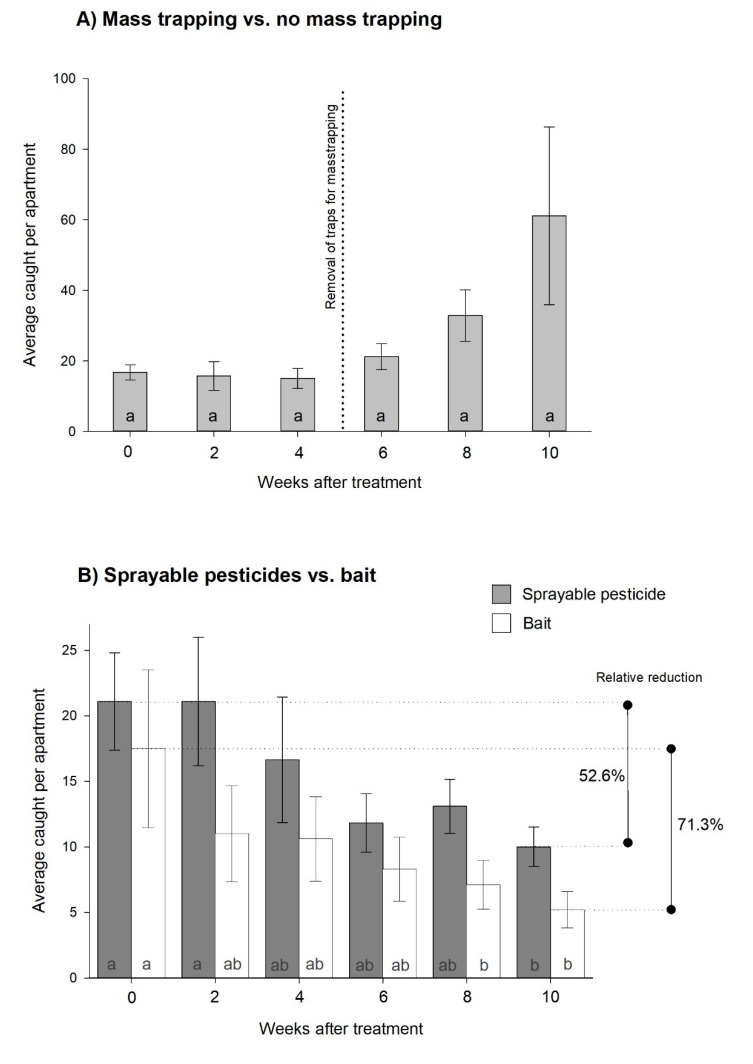
Effect of three control methods on the *Ctenolepisma longicaudata* population in 29 comparable apartments. (**A**) Effect of mass trapping with sticky traps before and after removal of trapsand (**B**) sprayable pesticide vs. poisoned bait. Different letters in the bars indicate a significant difference to other bars of the same color (Dunn’s method, *p* < 0.05).

Experiment 1, three different control strategies: The additional traps used for mass trapping captured an average of 22.5 ± 5.6 and 32.0 ± 9.4 individuals per apartment during the first and second fortnights, respectively. This produced a marginal decline of *C. longicaudata* (9.5%) in the monitoring traps, and the population increased to levels above the starting point when mass trapping was terminated (rm-ANOVA; χ2 = 11.4, *df* = 5, *p* = 0.044, Figure 1A). No significant differences were observed between weeks, even though there was significance in the overall test (all pairwise multiple comparisons *p* = 0.167).

No significant difference was observed between the spray and bait treatments, and both reduced the population significantly after 10 and 8 weeks, respectively (two-way rm-ANOVA; time: F = 6.7, *df* = 5, *p* < 0.001 and spray vs. bait: F = 2.2, *df* = 1, *p* = 0.154, Figure 1B). In total, permethrin reduced the initial population by 52.6%, whereas the bait yielded a 71.3% reduction during the 10 weeks of testing.

**Figure 2:**
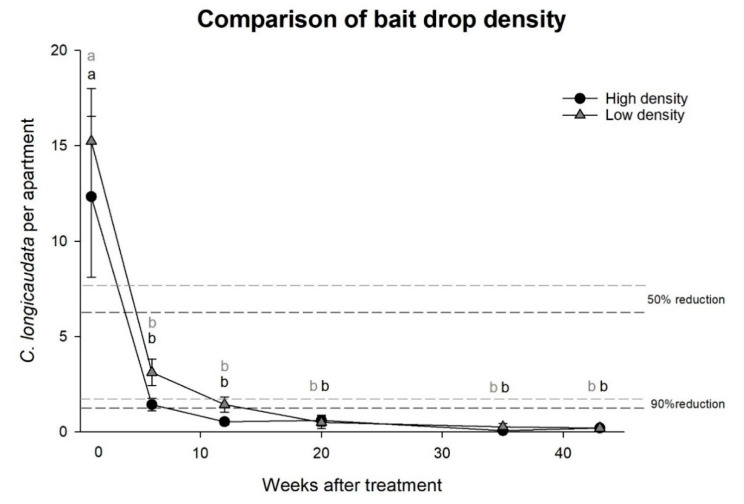
Effects of high or low density poisoned bait treatment on the *Ctenolepisma longicaudata* population. Different letters indicate significant differences between measurements (Dunn’s method, *p* < 0.05).

Experiment 2, effects of high or low density bait droplets: The cost-efficiency (i.e., time and amount of bait used per apartment) was found to be well within normal pest control operations as only 0.30 to 0.55 g bait and 10 to 15 min were spent per visited apartment (Table 2). With this approach, a significant reduction of more than 90% in the *C. longicaudata* populations in the apartments was observed with the high and low density bait droplets, and the effect through time was equally present in both treatments (two-way rm-ANOVA; time: F = 24.1, *df* = 5, *p* < 0.001 and high vs. low density: F = 0.4, *df* = 1, *p* = 0.516, Figure 2).

**Figure 3:**
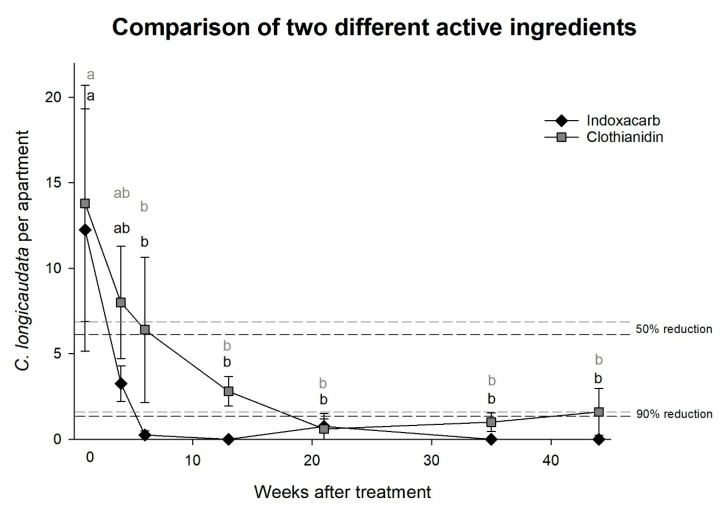
Effect of two different active bait ingredients on the *Ctenolepisma longicaudata* population size in apartments. Different letters indicate significant differences between measurements (Dunn’s method, *p* < 0.05).

Experiment 3, comparison between two different active ingredients: No significant difference was observed between indoxacarb and clothianidin, and both baits reduced the *C. longicaudata* population significantly after six weeks (two-way rm-ANOVA; time: F = 5.4, *df* = 6, *p* < 0.001 and indoxacarb vs. clothianidin: F = 0.9, *df* = 1, *p* = 0.380, Figure 3). Indoxacarb reduced the population size by 50% and 90% earlier than clothianidin and yielded three zero-measurements, while clothianidin failed to eliminate *C. longicaudata* during the test period.

**Figure 4:**
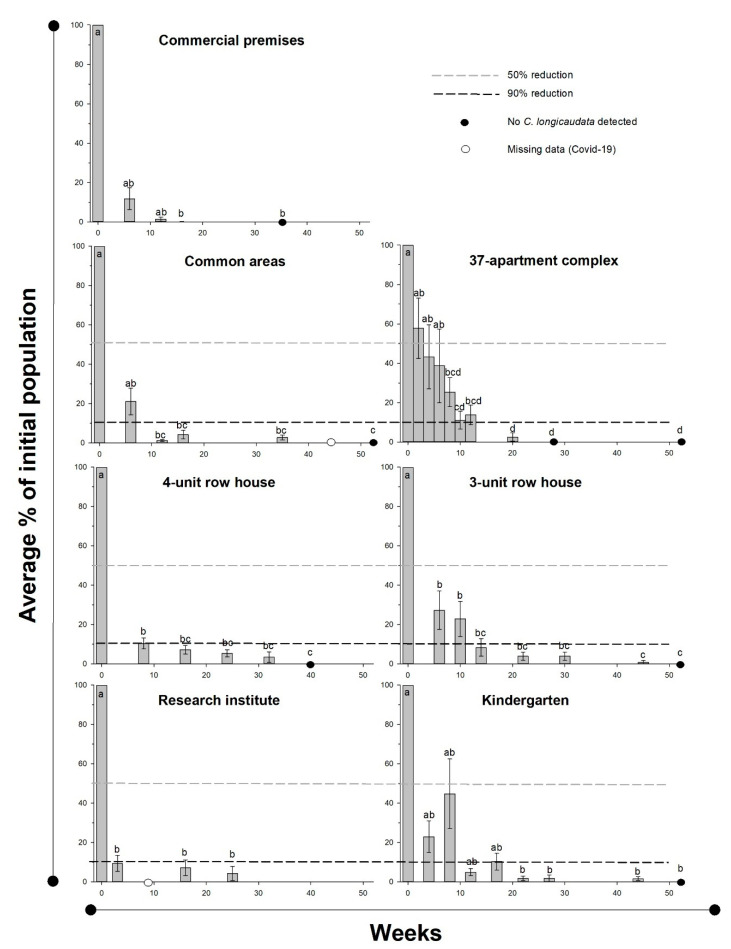
Effect of poisoned bait treatment on the *Ctenolepisma longicaudata* population in seven different localities. Different letters indicate significant differences between measurements in a locality (Dunn’s method, *p* < 0.05). Black circles indicate zero-measurement of individuals and white circles indicate missed samples due to Covid-19.

Experiment 4, field efficiency at seven different localities: Significant declines in *C. longicaudata* population densities were observed at all bait-treated localities (Table 4, Figure 4). A significant reduction in the population was observed across all localities after an average of 10.1 ± 2.2 weeks, and a 90% reduction was recorded after 17.0 ± 1.2 weeks. Six of the seven localities ended with zero-measurements. Only four small individuals were caught during the last collection at the locality without complete eradication, which was 25 weeks after applying the bait.

**Figure 5:**
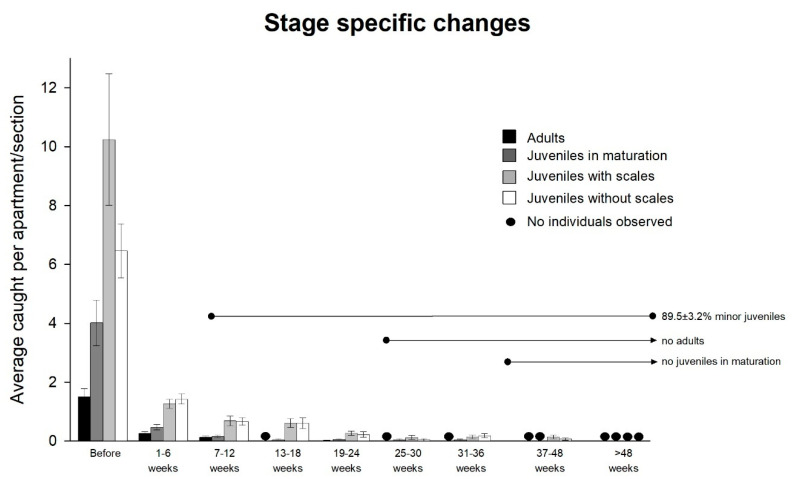
Changes in *Ctenolepisma longicaudata* developmental stages after the poisoned bait treatment. Data from seven different localities are pooled to show the general trends. Black circles indicate zero-measurement of a stage.

The trap catches prior to applying the bait (Experiment 4) showed a near building-wide distribution of *C. longicaudata*. In total, 158 of the 207 investigated rooms had *C. longicaudata*, and 58.2% of these rooms exhibited reproductive activity through the presence of small individuals without scales. A total of 39.9% of the infested rooms had at least three of the four life stage categories simultaneously. The demographics of the population changed during bait control at all locations (Figure 5). All stages declined initially, but the later measurements were strongly dominated by the small stages. The two minor stages comprised 89.5 ± 3.2% of the catch as of seven to 12 weeks; no adults were observed after 19 to 24 weeks, and no maturing juveniles were observed after 31 to 36 weeks.

**Figure 6:**
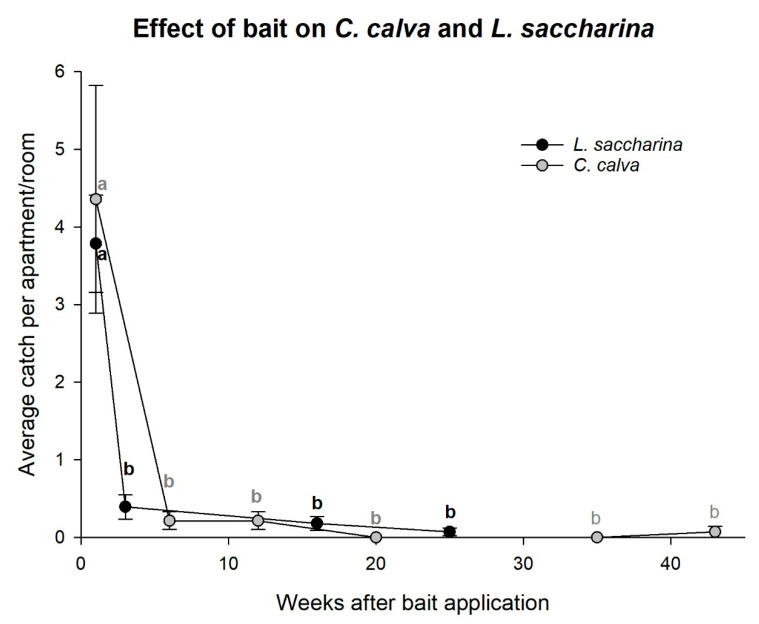
Effects of poisoned bait treatment on *Lepisma saccharina* and *Ctenolepisma calva* populations. Different letters indicate significant differences between measurements (Dunn’s method, *p* < 0.05).

*Ctenolepisma calva* and *L. saccharina* showed similar declines as seen for *C. longicaudata* and were significantly reduced after bait was applied for three to six weeks (rm-ANOVA; *C. calva*: χ2 = 56.57, *df* = 5, *p* < 0.001 and *L. saccharina*: χ2 = 65.95, *df* = 3, *p* < 0.001, Figure 6).

## 4. Discussion

This study shows a distinct effect from using bait to eradicate *C. longicaudata*. The application of several small droplets appeared to be a cost-efficient strategy for knock down and long-term eradication of this pest population. Several localities experienced an extended period of minor activity that delayed final eradication, but the demographic changes indicated that this was a result of a dying population maintained by eggs deposited early during management or by a few females who initially avoided the toxins.

Our initial experiment showed comparable effects from sprayable pesticides and bait. Other liquid pesticides, different application methods, elevated doses or repeated application may have yielded different results compared to our limited use of sprayable pesticides. Our progress in development of a control strategy was mainly based on improved residential and technician safety from the use of bait [19]. The initial experiment also used bait stations, which most likely reduced the effect compared to the more numerous, widely dispersed small bait droplets [6]. This observation indicates that the control effect from an adequately optimized bait strategy is likely to surpass the cautious use of a sprayable pesticide. Availability of pesticides and their indoor use are regulated by national or international legislation, and other control options may surely prove even more efficient than baits. However, Norwegian law discourages the use of sprayable pesticides to safeguard the indoor environment. Choice of strategy should always be in favor of the safest methods, which in this case is baits and not sprayable pesticides. We therefore also attempted to evaluate the intensity of baiting, i.e., droplet density, number of applications, odor stimulation for increased foraging, and concentration of the active ingredient (ant vs. cockroach bait). However, the difference between the seven localities with slightly different treatments was very limited, and minor distinctions between treatments were most likely masked by the strong overall population declines. Comparable effects appeared regardless of the intensity of the bait strategy, and the effects were equally present in commercial and office units as in large apartments or row houses. The method also appears to be effective against *L. saccharina* and *C. calva* as they died at comparable rates. Further studies are needed before final conclusions can be drawn regarding these species because there are distinct biological disparities between *C. longicaudata* and *L. saccharina*. The latter is strongly dependent on moist conditions [17,32] and can often be eradicated by drying out the habitat or through local bait- or spray treatment only [2,3].

It is interesting that bait has not been used against *C. longicaudata*. This species has received little research attention until its sudden and widespread increase in Norway [6,8] and its recent appearance in other European countries [10,11,12,13,14,15,16]. In this study, we deployed bait in a different way compared to more traditional cockroach treatments [27]. We were able to increase the probability of bait encounters to reach a large enough proportion of the population to tip the balance in favor of eradication by using many small droplets of widely distributed bait. This was in line with a Dutch study showing comparable effects from a similar strategy [16]. Indoxacarb, the main active ingredient used in this study, has delayed toxicity [33,34,35]. This may be a key factor for eradication by allowing a more whole-body distribution of the poison and allowing poisoned individuals to move into loose aggregations and hiding places before dying. We rarely observed dead individuals, although the control effect was strong. Dead conspecifics are used by *C. longicaudata* both as a protein source and most likely as a supply of beneficial symbionts [17,31]. A high degree of consumption of dead conspecifics may have promoted secondary poisoning and can be more crucial to the long-term effects compared to secondary poisoning during cockroach control [27,36,37]. The contribution from secondary poisoning is difficult to determine from direct effects in field studies, but this factor may be important in a protein-limited indoor environment. The clothianidin bait shows a limited level of secondary poisoning in the laboratory [30], but it appeared as efficient as indoxacarb in our field experiment. Multiple functional active ingredients [30] may be important to handle potential resistance problems in the future [38].

The persistent and comparable declines at all locations indicate an overuse of bait in several of our tests and highlight the small amount of bait needed to eliminate nuisance. *C. longicaudata*, which has a strong general affinity for sugars [17]. This species consumes bait dried-out under field conditions [30] and willingly feeds on dried sugar-treated paper [31]. Therefore, it is likely that *C. longicaudata* feed on the remains of dry bait and that a robust residual effect is present even when small amounts of bait are applied. All single bait treatments performed as well as the multiple bait applications. Even in the kindergarten, where bait was not continuously present, but removed after two brief periods, it still maintained its effect for almost a year. This also indicates a strong contribution from secondary poisoning and highlights the importance of precise baiting focused on a wide distribution with a high probability of bait encounters. Such an approach appears to release the desired control effects and promote successful eradication regardless of reapplication frequency. This contrasts with cockroach baiting, where multiple applications are common to ensure the freshness of baits and sustain the control effect [27,28]. A second treatment may be applied for silverfish bait control at a much later stage than we did in our studies to counter the potential loss of effect from removing bait by cleaning.

The interactions among habitat preferences, nutritional demands, and spatial distribution should be considered to understand the mechanisms of a successful bait strategy. Cockroaches have a lifecycle comparable to *C. longicaudata* [1]. They are partially restricted to areas with both food and favorable conditions [2,3,9]. This makes them susceptible to a bait strategy, as the foraging pattern and location of harborages can be predicted [27]. Little is known about the spatial distribution and foraging in *C. longicaudata*, but they appear to have few habitat restrictions indoors [1]. Energy sources are available in surplus through carbohydrates found in dry leftovers, plant-based materials, and even paper [2,17,39], whereas amino acids, lipids, and other crucial nutritional elements needed for growth, hormone production, egg development, and other complex biochemical processes [40,41] may be in limited supply. Easy access to energy may support a building-wide spatial distribution, and an active search for restricted nutrition might fortify this situation as new suitable parts of the building may be made available during foraging. In this study, we observed a uniform distribution of *C. longicaudata* with all life stages in most parts of the infested building. This distinguishes *C. longicaudata* from cockroaches, which depend more strongly on aggregations for access to food, survival, and reproduction [1,9,27]. This finding also helps explain the success with the widely distributed small bait droplets. When *C. longicaudata* are using a larger proportion of a building, it is also reasonable to assume that some hidden enclaves may maintain low infestation levels for some time due to avoidance of the bait. Such a pattern was observed in our study through a long period of minor activity. However, when resources at such hidden locations are depleted, the probability of encountering bait will increase through a forced nutritional search, and finally full eradication success is realized.

The bait approach is highly cost efficient when considering the low dose needed (less than 1 g per 100 m^2^) and the short time the technicians spent in each apartment (10–15 min per visit). Depending on the size of the building and the number of stakeholders involved, the time used to organize and prepare the whole-building approach may surpass technical treatment time. Strong control effects were achieved with as little bait as 0.5 to 1.0 g per 100 m^2^ by using professional pest control technicians, with proper training in bait placement and knowledge of *C. longicaudata* biology. Even compared to our restricted use of sprayable pesticides, this promotes safety when combined with the low concentration of active ingredients in the bait. Additionally, applying as much as 30 g of bait per apartment of the same type of bait used in this study did not leave toxic dust residues in a cockroach study [42]. This approach might even be considered safe with children and pets if applications are made cautiously at inaccessible locations in combination with necessary precautionary information to residents.

Norway has experienced large problems with *C. longicaudata* compared to the rest of the world, and there is a dominance of the problem in newer buildings [6]. These differences may arise from variations in trade systems, introduction patterns, indoor environmental conditions, or vernacular architecture. *C. longicaudata* has likely been introduced on multiple occasions through international trade goods, and now spreads through domestic invasion paths to private homes in Norway [6]. We studied and approached the current problem from the end of the invasion path, but as bait appeared to be efficient in all environments tested, including the parking house and storage facilities, it would be beneficial to direct control efforts towards the potential dispersal sources. Pesticide use in private homes could be prevented by avoiding introductions all together if source populations of *C. longicaudata* at international and domestic trade hubs or storage facilities were managed.

## 5. Conclusions

Poisoned bait succeeded in eradication of *C. longicaudata* infestations in urban environments. This study therefore demonstrates a low risk and low-cost approach towards a solution, and although the final and full eradication effect was progressing slowly at the end, it appeared persistent across multiple indoor habitats. Other European countries also report growing problems with this nuisance pest [11,12,13,14,15,16] and may now adopt this approach to prevent excessive use of pesticides indoors.

## Figures and Tables

**Table 1:** Similarities among the apartments tested for mass trapping (sticky traps; Trapper monitor and insect glue traps), sprayable pesticides (9.5 g/kg Permethrin and 3.4 g/kg Pyrethrin II) and poisoned bait (Indoxacarb; 0.05%) against *Ctenolepisma longicaudata*. Averages are given with ± SEM.

Control Method	^#^ of Replicates	Apartment Size (m^2^)	^#^ of Residents	Messy/Tidy ^†^	Dirt (g) ^‡^	Initial Population	Temperature (°C)	Humidity (% RH)
Mass trapping	n = 8	80.8 ± 5.2	2.3 ± 0.3	3/5	44.8 ± 1.9	16.8 ± 2.2	23.8 ± 0.2	38.9 ± 1.5
Spray pesticides	n = 11	81.9 ± 8.1	2.1 ± 0.3	3/8	41.0 ± 1.4	21.1 ± 3.7	22.8 ± 0.3	38.8 ± 1.1
Bait	n = 10	85.6 ± 5.6	1.6 ± 0.3	3/7	42.9 ± 1.0	17.5 ± 6.0	23.5 ± 0.3	40.3 ± 1.0

^†^ = Messy; large amounts of furniture, objects, and clutter, Tidy; little furniture, objects, and clutter; ^‡^ = amount collected by standardized and timed vacuum cleaning; ^#^ = number.

**Table 2:** Bait amount (0.6% indoxacarb), droplet size, and time used by the pest control technician per visit/treatment in a building-wide experiment against *Ctenolepisma longicaudata*. High density and low-density bait treatments were compared in a high-rise housing cooperative.

Experimental Details	High Density	Low Density
Number of apartments:	84	88
Size of apartments:	40–140 m^2^	40–140 m^2^
Bait used per apartment:	0.55 g	0.30 g
Droplet size applied:	0.009 g	0.008 g
Time spent per apartment:	15 min	10 min
Estimated time for organization:	2–3 h total ^†^	

^†^ = Time for organization equal regardless of droplet density.

**Table 3:** Description of the treatment and the population evaluation by sticky traps of Ctenolepisma longicaudata in seven different localities with Advion^®^ Cockroach (0.6% indoxacarb) or Advion^®^ Ant (0.05% indoxacarb).

Location (Habitat)	Bait (Active Ingredient)	Number of Application	Droplet Density	Fortnights with Evaluation Traps	Separation of Fortnights	Trap Density or Number
**Commercial premises**	0.6% indoxacarb	1	Low	5	4 to18 weeks	327 traps/11 zones
**Common areas**	0.6% indoxacarb	2	Low	7	4 to 18 weeks	146 traps/5 zones
**37-apt. complex**	0.6% indoxacarb	2 ^†^	High	10	0 to 26 weeks	0.32-0.22 traps/m^2^
**4-unit row house**	0.05% indoxacarb	3	High	6	8 weeks	0.25 traps/m^2^
**3-unit row house**	0.6% indoxacarb	1 ^‡^	High	8	4 to 15 weeks	0.12 traps/m^2^
**Research institute**	0.6% indoxacarb	1	Low	5	2 to 8 weeks	147 traps/47 rooms
**Kindergarten**	0.06% indoxacarb	2 ^§^	High	9 ^¶^	4 to 16 weeks	0.3 traps/m^2^

^†^ = only partial 3rd treatment, ^‡^ = supported by bait stations, ^§^ = bait removed after 5 and 22 days, ^¶^ = traps only present on weekends.

**Table 4:** Tests of field efficiency of poisoned bait (0.6% indoxacarb) on *Ctenolepisma longicaudata* at seven different locations. Test statistics (repeated-measures ANOVA), week of first significant reduction of the population, week of more than 90% reduction of the population, and week of total elimination of the population. Averages are given with ± SEM.

Location	χ^2^ or F/, *p*-Value	Week of First Significant Reduction (*p* < 0.05)	Week > 90% Reduction	Zero-Measurement
Commercial premises	χ^2^ = 15.2, *p* = 0.004	16 weeks	16 weeks	Yes, week 35
Common areas	χ^2^ = 39.3, *p* < 0.001	12 weeks	12 weeks	Yes, week 55
37-apartment complex	χ^2^ = 102.9, *p* < 0.001	8 weeks	20 weeks	Yes, week 28
4-unit row house	F = 375.2, *p* < 0.001	8 weeks	16 weeks	Yes, week 40
3-unit row house	F = 46.8, *p* < 0.001	6 weeks	14 weeks	Yes, week 52
Research institute	χ^2^ = 66.0, *p* < 0.001	3 weeks	16 weeks	No, week 25 ^†^
Kindergarten	χ^2^ = 35.7, *p* < 0.001	22 weeks	22 weeks	Yes, week 53
	Average:	10.1 ± 2.2 weeks	17.0 ± 1.2 weeks	

^†^ = experiment still running at time of manuscript submission.

## References

[1] Robinson W.H. (2005). Urban Insects and Arachnids—A Handbook of Urban Entomology.

[2] Mallis A., Hedges S.A., Moreland D. (2011). Handbook of Pest Control: The Behaviour, Life History, and Control of Household Pests.

[3] Bennett G.B., Owens J.M., Corrigan R.M. (2010). Truman’s Scientific Guide to Pest Management Operations.

[4] Querner P. (2015). Insect pests and integrated pest management in museums, libraries and historic buildings. Insects.

[5] Szpryngiel S. (2018). Långsprötad Silverfisk i Museer, Bibliotek och Arkiv i Sverige.

[6] Aak A., Rukke B.A., Ottesen P.S., Hage M. (2019). Long-Tailed Silverfish (Ctenolepisma Longicaudata)—Biology and Control.

[7] Schoelitsz B., Brooks M. Distribution of *Ctenolepisma longicaudata* (Zygentoma: Lepismatidae) in the Netherlands. Proceedings of the Eighth International Conference on Urban Pests.

[8] Hage M., Rukke B.A., Ottesen P.S., Widerøe H.P., Aak A. (2020). First record of the four-lined silverfish, *Ctenolepisma lineata* (Zygentoma, Lepismatidae), in Norway, with notes on other synanthropic lepismatids. Nor. J. Entomol..

[9] Bonnefoy X., Kampen H., Sweeney K. (2008). Public Health Significance of Urban Pests.

[10] Pape T., Wahlstedt U. (2002). En silverborstsvans nyinförd till Sverige (Thysanura: Lepismatidae). Entomol. Tidskr..

[11] Thomsen E., Kongsstovu S.I., Dahl H.A., Mikalsen S.O. (2019). *Ctenolepisma longicaudata* (Escherich, 1905): A common, but previously unregistered, species of silverfish in the Faroe Islands. BioInvasions Rec..

[12] Kulma M., Vrabec V., Patoka J., Rettich F. (2018). The first established population of the invasive silverfish *Ctenolepisma longicaudata* (Escherich) in the Czech republic. BioInvasions Rec..

[13] Goddard M.R., Foster G.J., Holloway G.J. (2016). *Ctenolepisma longicaudata* (Zygentoma: Lepismatidae) new to Britain. Br. J. Ent. Nat. Hist..

[14] Meineke T., Menge K. (2014). Ein weiterer fund des Papierfischens *Ctenolepisma longicaudata* Escherich, 1905 (Zygentoma, Lepismatidae) in Deutchland. Entomol. Nachr. Ber..

[15] Nierop B.M.B., Hakbijl T. (2002). *Ctenolepisma longicaudatum* heeft ongemerkt bebouwn Nederland veroverd. Entomolog. Ber..

[16] Gutsmann V. (2019). Ein “Fischköder” der besonderen Art. DPS Fachz. Schädlingsbekämpfung.

[17] Lindsay E. (1940). The biology of the silverfish, *Ctenolepisma longicaudata*, with particular reference to its feeding habits. Proc. Roy. Soc. Vic..

[18] Butte W., Plüschke P. (2004). Sources and impacts of pesticides in indoor enviroments. Indoor Air Pollution.

[19] Dhang P. (2011). Urban Pest Managment—An Environmental Perspective.

[20] Oudejans L., Mysz A., Gibb Snyder E., Wyrzykowska-Ceradini B., Nardin J., Tabor D., Starr J., Stout D., Lemieux P. (2020). Remediating indoor pesticide contamination from improper pest control treatments: Persistence and decontamination studies. J. Hazard. Mater..

[21] Dhang P. (2014). Urban Insect Pests—Sustainable Managment Strategies.

[22] Dhang P. (2016). Innovations in insect baiting and its role in reducing insecticide load in urban pest control. Int. Pest Control..

[23] Anikwe J.C., Adetoro F.A., Anogwih J.A., Makanjuola W.A., Kemabonta K.A., Akinwande K.L. (2014). Laboratory and field evaluation of an indoxacarb gel bait against two cockroach species (Dictyoptera: Blattellidae, Blattidae) in Lagos, Nigeria. J. Econ. Entomol..

[24] Appel A.G. (2003). Laboratory and field performance of an indoxacarb bait against German cockroaches (Dictyoptera: Blattellidae). J. Econ. Entomol..

[25] Rabito F.A., Carlson J.C., He H., Werthmann D., Schal C. (2017). A single intervention for cockroach control reduces cockroach exposure and asthma morbidity in children. J. Allergy Clin. Immunol..

[26] Matos Y.K., Schal C. (2016). Laboratory and field evaluation of Zyrox fly granular bait against Asian and German cockroaches (Dictyoptera: Blattellidae). J. Econ. Entomol..

[27] Tee H.S., Lee C.Y., Dhang P. (2014). Sustainable cockroach managment using insecticidal baits: Formulations, behavioural responses and issues. Urban Insect Pests—Sustainable Managment Strategies.

[28] Wang C., Eiden A., Cooper R., Zha C., Wang D.S. (2019). Effectiveness of building-wide integrated pest management programs for German cockroach and bed bug in a high-rise apartment building. J. Integr. Pest Manag..

[29] Sims S.R., Appel A.G. (2012). Efficacy of commercial baits and new active ingredients against firebrats and silverfish (Zygentoma: Lepismatidae). J. Econ. Entomol..

[30] Aak A., Hage M., Rukke B.A. (2020). Long-tailed silverfish (*Ctenolepisma longicaudata*) control; bait choice based on primary and secondary poisoning. Insects.

[31] Woodbury N., Moore M., Gries G. (2013). Horizontal transmission of the microbial symbionts *Enterobacter cloacae* and *Mycotypha microspora* to their firebrat host. Entomol. Exp. Appl..

[32] Sahrhage D. (1954). Ökologische untersuchungen am Ofenfischchen, *Thermobia domestica* (Packard), und Silberfischchen, *Lepisma saccharina* L.. Z. Angew. Entomol..

[33] McCann S.F., Annis G.D., Shapiro R., Piotrowski D.W., Lahm G.P., Long J.K., Lee K.C., Hughes M.M., Myers B.J., Griswold S.M. (2001). The discovery of indoxacarb: Oxadiazines as a new class of pyrazoline-type insecticides. Pest Manag. Sci..

[34] Wing K.D., Sacher M., Kagaya Y., Tsurubuchi Y., Mulderig L., Connair M., Schnee M. (2000). Bioactivation and mode of action of the oxadiazine indoxacarb in insects. Crop Prot..

[35] Gondhalekar A.D., Nakayasu E.S., Silva I., Cooper B., Scharf M.E. (2016). Indoxacarb biotransformation in the German cockroach. Pest. Biochem. Physiol..

[36] Buczkowski G., Scherer C.W., Bennett G.W. (2008). Horizontal transfer of bait in the German cockroach: Indoxacarb causes secondary and tertiary mortality. J. Econ. Entomol..

[37] Bayer B.E., Pereira R.M., Koehler P.G. (2012). Differential consumption of baits by pest blattid and blattellid cockroaches and resulting direct and secondary effects. Entomol. Exp. Appl..

[38] Zhu F., Lavine L., O’Neal S., Lavine M., Foss C., Walsh D. (2016). Insecticide resistance and management strategies in urban ecosystems. Insects.

[39] Pothula R., Shirley D., Perera O.P., Klingeman W.E., Oppert C., Abdelgaffar H.M.Y., Johnson B.R., Jurat-Fuentes J.L. (2019). The digestive system in Zygentoma as an insect model for high cellulase activity. PLoS ONE.

[40] Chapman R.F., Simpson S.J., Douglas A.E. (2013). The Insects—Structure and Function.

[41] DeVries Z.C., Appel A.G. (2014). Effects of temperature on nutrient self-selection in the silverfish *Lepisma saccharina*. Physiol. Entomol..

[42] Wang C., Eiden A., Cooper R., Zha C., Wang D., Reilly E. (2019). Changes in indoor insecticide residue levels after adopting an integrated pest management program to control German cockroach infestations in an apartment building. Insects.

